# Challenges of caregivers providing care to children with disabilities at non-governmental organisations in Tshwane townships, South Africa

**DOI:** 10.4102/ajod.v11i0.930

**Published:** 2022-07-28

**Authors:** Sharifa Moosa-Tayob, Patrone R. Risenga

**Affiliations:** 1 Department of Health Studies, College of Human Sciences, School of Social Sciences, University of South Africa, Pretoria, South Africa; 2 Department of Occupational Therapy, School of Health Care Sciences, Sefako Makgatho Health Sciences University, Pretoria, South Africa

**Keywords:** non-governmental organisations, caregivers, children with disabilities, caregiver challenges, Tshwane townships

## Abstract

**Background:**

Caregivers of children with disabilities are vital stakeholders when it comes to safeguarding the health, well-being and overall survival of the children that they care for. Caregivers, however, face many challenging conditions that make it difficult for them to optimally fulfil their caregiving role. Understanding these challenges is crucial for developing empowerment programmes for caregivers, which will ensure that children with disabilities receive comprehensive, optimal care and that caregivers experience a good quality of life.

**Objectives:**

The aim of this study was to explore and describe the experiences of caregivers providing care to children with disabilities at non-governmental organisations (NGOs) in townships of Tshwane, South Africa.

**Method:**

This study followed an exploratory, descriptive and contextual research design within a qualitative methodology. The population in this study included caregivers who care for children with disabilities at NGOs (*n* = 10). Participants for the study were selected using the purposive sampling technique. Data were collected by conducting semistructured interviews with caregivers. Data were analysed according to the six phases of thematic analysis proposed by.

**Results:**

The study revealed six themes that represent the challenges experienced by caregivers, namely (1) initial impressions, (2) rendering care, (3) stress, (4) lack of outside support, (5) coping and (6) poor community recognition.

**Conclusion:**

Support from the Departments of Health and Social Development and other institutions providing community rehabilitation services to townships should be provided to caregivers in order to empower them with skills and knowledge to effectively address the challenges they face so that they can render optimal care to the children they care for.

**Contribution:**

Results of this study could assist in ensuring improved recognition, resilience and supportive resources for caregivers in collaboration with community based rehabilitation stakeholders in the community that would ultimately lead to improved care for children with disabilities in townships within South Africa.

## Introduction

South Africa is amongst the 193 countries that have taken on the responsibility of fulfilling their responsibilities under the Convention on the Rights of Persons with Disabilities. Included in this responsibility is to ensure that all children, including children with disabilities, enjoy the rights afforded to them without any discrimination (United Nations Children’s Fund [UNICEF] 2013). According to the United Nations (UN), approximately 250 million of the 600 million individuals globally who have a disability are children, and approximately 10% of children have a disability where long-term caregiving and prolonged access to health care are needed (UNICEF 2013). The global prevalence of disabilities ranges between 12% and 15%, with a distinct difference between developed and developing countries (Mitra & Sambamoorthi 2014). Most low-income countries have limited services available to families and children with disabilities, which results in many children with disabilities being neglected or underserved (Oskoui et al. 2013). According to the World Health Organization (WHO) and World Bank (2011), globally, 1 billion people have some type of disability, with 80% of people with disabilities residing in developing countries. Most disabilities are lifelong disorders, which therefore have no cure (Bolster et al. 2017). There are, however, various interventions available which lessen the effect of disabilities and improve the quality of life of those affected by disabilities (Bolster et al. 2017).

Accurate data on the prevalence of children with disabilities in South Africa are difficult to establish; however, data that were last gathered during the 2011 national consensus revealed that approximately 2.1 million children in South Africa have a disability, thus making the prevalence of children with disabilities in South Africa to be 11.2% (Statistics South Africa [STATS SA], 2014). The highest prevalence of children with disabilities in South Africa was found in the age group 0–4 years, as 28.0% of children in this category were found to have a disability. The category of children aged 0–9 years had a prevalence of disability that was less than half of those in the 0–4 years category, as 10.0% of children in this age category were classified as having a disability (STATS SA, 2014).

In developing countries, many children with disabilities are cared for by caregivers in areas that are underdeveloped and have restricted access to training and skills development to optimally care for them (Bizzego et al. 2020). This results in a situation where children with disabilities receive inadequate care that directly affects their functional performance.

The term ‘NGO’ was created by the UN when they desired to consult with non-profit organisations (NPOs) and the private sector, who were not dependent on the government. The UN defines a non-governmental organisation (NGO) as:

[*A*]ny non-profit, voluntary citizens’ group which is organized on a local, national or international level. Task-oriented and driven by people with a common interest, NGOs perform a variety of services and humanitarian functions, bring citizens’ concerns to governments, monitor policies and encourage political participation at the community level. (Teegen, Doh & Vachani 2004: 466)

Mostashari (2005) categorises NGOs into two groups: (1) non-governmental organisations that have to acquire resources to sustain the programmes they run and (2) NGOs that take on the main role of advocacy and are governed by a board that has the function of capacity-building and development of management and governance tools.

According to Statistics South Africa (2014), South Africa had a registered number of 127 000 registered NGOs and 50 000 unregistered NGOs. Non-governmental organisations in South Africa differ in size and in the services they offer. To function effectively, NGOs have to collaborate with the government and other stakeholders. The Centre for Child Law (2017) states that NGOs for children with disabilities offer value services to children who are differently abled; however, these NGOs are not equipped with adequate resources to provide quality care.

A caregiver can be any individual who renders care to a person who is unable to care for himself or herself. Caregivers can provide services that are either formal or informal. Informal services are those services rendered by caregivers that are not remunerated. Formal services provided by caregivers are remunerated and can be provided to anyone who has psychological, physical or developmental needs (Musich et al. 2018).

Capri et al. (2018) adds that a caregiver is one who supports care recipients in performing their basic activities of daily living. Li and Song (2019) categorise caregivers into two main categories, namely formal caregivers and informal caregivers. Formal caregivers receive remuneration for their services and are employed by individuals or institutions such as an NGO. Informal caregivers provide care to care recipients without being remunerated. A prerequisite for formal caregivers is that they have to have received some training in their field of practice. Ku, Liu and Wen (2013) view formal caregivers as health care professionals. The caregiver role is a role with numerous duties and obligations towards the care recipient. The Alzheimer’s Society of York (2018) states that the caregiver role could be very overwhelming as it is active, intense and encompassing of many duties. This is supported by Walga (2019), who found the caregiving role to be very demanding as all the tasks expected by caregivers to implement place a great deal of responsibility on the caregivers. Geiger (2012) defines the basic roles for caregivers who care for children with disabilities as major and minor basic roles. Major roles entail caring for the children’s basic essential needs such as feeding, bathing and giving medication. Minor roles include stimulation and exercise to ensure optimum development (Geiger 2012). Science Care (2015) acknowledges that the caregiving role is demanding as it involves countless roles and responsibilities. It includes providing basic care, to care recipients and the administering of medication.

The act of caregiving could be a very rewarding experience for caregivers and could also be an experience that results in undesirable consequences for the caregiver (Jones et al. 2011). Caregiver burden is described as the emotional, physical, social and financial implications of providing care to people with disabilities and illness (Diameta et al. 2018). Caring for children with disabilities places persistent psychological and physical demands on the caregiver, which could result in the caregiver experiencing high levels of stress. As a direct result of caregiving, caregivers providing care to children with disabilities experience many stressors; however, they lack the ability to apprise and cope with stressors, which has a negative effect on the caregivers’ physical and mental health and overall functioning. The psychological stressors caregivers experience often manifest themselves not only in psychological problems but in physical problems as well, which all affect the quality of life for these caregivers. The caregiving role often causes physical and psychological stress for the caregiver. Theofilou (2012) explored the psychological effects of caregiving and found that caregivers experience symptoms such as depression, anxiety, guilt, anger, insomnia, generalised muscle pain and headaches. Phillips et al. (2016) infers that caregivers who are in a poor physical and psychological state are at a higher risk of providing poor quality care to care recipients.

Recognition of caregivers by community members is regarded as an extrinsic reward to caregivers (Akintola 2010). Caregivers being undermined by the community and not receiving the recognition they deserve cause caregivers’ discontent (Schneider 2020). Caregivers need inclusive programmes that empower them to render effective care to the individuals for whom they care. It is therefore crucial that caregivers receive recognition and support to render optimal care for children with disabilities (The Alzheimer’s Society of York 2018).

Caregiver preparedness denotes the readiness of a caregiver for carrying out all the caregiving tasks related to their caregiving role. Caregiver preparedness has a strong link to caregivers feeling less anxious and burdened and could assist with overcoming the negative aspects of caregiving. Musich et al. (2018) added that caregiver preparedness increases positive feelings such as hope and leads to better overall caregiver health. Norinder, Goliath and Alvariza (2017) suggest that caregivers who feel they are prepared provide better care to their care recipients. A strategy that could be used to improve caregiver effectiveness is understanding and improving caregivers’ level of readiness to start rendering care to care recipients (Marx et al. 2019). Lutz, Young, Creasy, Martz, Eisenbrandt, Brunny and Cook (2016) list numerous factors that influence the readiness of caregivers to assume the caregiver role. These factors include the presentation of the care recipient, the characteristics of the caregiver, caregiver knowledge, skills and availability of resources to carry out their caregiving duties. Caregivers have to be allocated the reagent resources if they are to render optimal caregiving services to children with disabilities (Soni et al. 2020). In addition to caregiver skills, Soni et al. (2020) found that a lack of resources posed a further challenge to the provision of optimum quality care to children who are differently abled. According to World Health Organization, International Labour Office & UNESCO (2005), governments are willing to accept and implement Community Based Rehabilitation (CBR) programmes at the national level. This is challenging as many countries lack resources to implement and sustain CBR programmes. Lack of resources therefore is a challenge on both the macro level and the micro level (NGOs) as well. The micro level is the level in which NGOs fall into.

The high number of disempowered caregivers is affirmed in the Framework and Strategy for Disability and Rehabilitation Services in South Africa (National Department of Health 2015), which highlights the high number of NGOs who have untrained caregivers looking after children with disabilities. The provision of education, resources and self-awareness is seen as an empowerment process, giving great power to its recipients (Elphick 2017). The concept of empowerment is based on the impression that it is possible to help people to cope and feel better through discourse and reflection between the professional and the client in need as well as the caregiver. Hage and Lorensen (2005) argue that by implementing an empowerment strategy, caregivers are given the opportunity to expose their weaknesses and limitations that ultimately help them to effectively come up with a strategy to effectively care for themselves and others. Numerous authors agree that programmes that help caregivers to change their behaviours in some positive way, helping caregivers to find resources within and outside the client and helping caregivers with the adjustment into the caring situation are all ways of helping caregivers reach the point of feeling empowered (Elphick 2017; Freid 2018; Hage & Lorensen 2005). Caregivers who feel empowered therefore provide better care to children who are differently abled.

## Statement of the research problem

Despite numerous research studies confirming the crucial role NGOs for children with disabilities and caregivers of children with disabilities play in providing and offering support to various stakeholders of children with disabilities such as families, communities and society; caregivers’ individual needs are often overlooked, disregarded and misunderstood. In short, literature pertaining to the challenges caregivers experience strongly suggests that the majority of caregivers at NGOs for children with disabilities do not feel empowered and therefore emphasises the need for caregiver empowerment. Caregivers of children with disabilities often feel disempowered as they lack the skills and knowledge to provide optimal care to their care recipients (Zuurmond et al. 2019). The challenges of caregivers at NGOs for children with disabilities need to be investigated in order to guide caregivers in rendering optimal care to children with disabilities at NGOs as well as guide relevant CBR stakeholders in providing ideal support to caregivers at NGOs.

## Purpose of the study

The data presented here were part of a larger study titled ‘A programme to empower caregivers of children with disabilities at non-governmental organisations’ that aimed to develop a programme to empower caregivers who care for children with disabilities at NGOs with skills and knowledge to effectively address the challenges they face in their caregiving role. The specific objective of this paper is to explore and describe the challenges of caregivers providing care to children with disabilities at NGOs.

## Research methodology

This study made use of a qualitative research design that is exploratory, descriptive and contextual in nature. This approach was used as the researcher sought to explore, describe and understand the meanings that individuals or groups attribute to human and social phenomena (Creswell & Plano Clark 2017). A detailed understanding of caregivers’ challenges was established by interviewing caregivers in the context where services are rendered. Semistructured interviews are used when the researcher has a list of predetermined questions regarding the research objectives in order to obtain information-rich responses for participants. The interview schedule contained specific questions for caregivers that related to challenges caregivers experienced when rendering care to children with disabilities. Possible probes were also included in each interview schedule.

## Study setting

This study was conducted at various selected NGOs that provide care to children with disabilities in the townships of Tshwane. Tshwane is a metropolitan municipality in the northern side of Gauteng province, South Africa. According to the City of Tshwane’s official website (last updated in 2015), there are over 200 health care NGOs registered with the City of Tshwane. Of these registered health care NGOs, approximately 30 NGOs within townships cater for children with disabilities (City of Tshwane 2015). Children cared for by these NGOs are children with moderate to severe disabilities such as cerebral palsy, severe intellectual disability, autism and spina bifida. Interviews were conducted at caregivers’ natural settings. Burns, Grove and Gray (2015) define a natural setting as a setting where the environment in which the study is being conducted is not manipulated by the researcher. Caregivers were interviewed at the premises of the NGO where they are employed.

## Study population

The population comprised female caregivers aged 18 years and up, employed at NGOs to render direct care to children with disabilities. All caregivers had been employed at the NGO for at least 6 months, as NGO managers stated that it took at least 6 months for caregivers to gain relevant experience.

## Sampling technique

The number of participants in qualitative studies is generally small; therefore, nonprobability, nonrandom sampling methods are used (Kumar 2014). A purposive sampling technique was selected to ensure that only participants who have the required characteristics for the study were selected. The caregivers were selected from NGOs who render direct care to children who are disabled, in order to purposefully inform an understanding of the phenomenon in the study and the problems that are central to the research.

## Sample size

It is not feasible to provide definite sample sizes; however, the number of participants in the study was determined by data saturation.

Saturation is described as the process the researcher uses to gather and analyse data up to the point where new insights are no longer observed (Polit & Beck 2017). The data saturation point was reached after interviewing 10 caregivers from four NGOs, when the researcher stopped gathering new information from participants.

## Data collection

The researcher collected data through conducting semistructured interviews in English with caregivers from June 2020 to November 2020; an additional caregiver was interviewed in February 2022. Interviews were audio recorded using an audio tape-recorder. The duration of interviews was between 45 and 90 min. The interview scheduled required pseudonyms, dates, respondent numbers and biographical information for each participant. Finally, the interview schedule contained specific questions for caregivers related to the research objectives. Questions were related to the daily experiences of caregivers when rendering care to children with disabilities. Probes were also included in each interview schedule.

## Data analysis

Data were analysed simultaneously with data collection. This simultaneous process of data collection and data analysis were done to enable the authors to develop an understanding about the phenomenon in question and aided the researcher in determining when data saturation was reached. Data were analysed according to the six phases of thematic analysis proposed by Braun and Clarke (2006) (in Jackson, McDowall, Mackenzie-Savvy & Whiting 2016), which are as follows:

*Phase 1: Familiarisation* – This phase entailed the researcher carefully reading through the transcripts so that the researcher could gain a deeper understanding into the meaning of the transcripts.

*Phase 2: Coding* – In this phase of data analysis, the researcher produced initial codes for the data collected. Patterns were identified in the data by grouping data sets that were alike.

*Phase 3: Searching for themes* – During this phase of data collection, the researcher generated themes by extracting, sorting and grouping relevant codes.

*Phase 4: Reviewing themes* – This phase involved a deeper review of the identified themes. Themes were checked in relation to the coded extract.

*Phase 5: Defining and naming themes* – This step captured the core of what each theme consisted of. This phase enabled the researcher to clearly identify what the themes were and what they were not.

*Phase 6: Writing the report* – The final phase of data analysis involved writing up the report by means of tables and figures in the form of word-for-word quotes that were used to support themes and subthemes. The researcher ensured the report was succinct, clear, rational and nonrepetitive and offered interesting accounts of the stories the data told.

The authors made use of an inductive approach to analyse data. Creswell and Plano Clark (2014) refer to inductive reasoning as a bottom-up approach where the researcher uses information gathered from participants to generate themes and then interconnect those themes to generate theory. The authors generated themes constructed from the objectives of the study as well as linked two or more concepts introduced by interviewees into one group, reasoning how they might go together as they were formulated through units of meaning. To facilitate the retrieval of what was said on each topic, data were coded, which were marked on a copy of the transcript from a word or phrase that represented what the researcher thought the given participant’s response meant. To generate themes with the supporting subthemes, 10 caregiver transcripts were analysed.

## Trustworthiness

Trustworthiness was ensured through credibility, dependability, conformability and transferability. Credibility was ensured through using an audio recorder to record the semistructured interviews. To ensure dependability, the research procedures and the process employed during the study have been documented in detail in order to enable future researchers to repeat the study. Transferability related to the external validity of the research denotes to what extent the findings of the research can be generalised to other people, contexts, times and outcomes (Yin 2016). Although the findings of this project related to the context of where the research was conducted and not to all caregivers who provide care to children with disabilities at NGOs, the authors ensured external validity by conducting the research in a real-life setting, the NGOs where the care caregivers were employed. The study was thus conducted at the NGOs where caregivers were employed.

### Ethical considerations

The authors conformed to the World Medical Association Declaration of Helsinki Ethical Principles for Medical Research Involving Human Subjects (World Medical Association General Assembly 2013). Ethical clearance was obtained from the University of South Africa’s Health’s Studies Research Ethics Committee (HSHDC/975/2020). Non-governmental organisation managers granted the researcher written permission to conduct the study at their facilities. Before interviews were conducted, the participants who had indicated that that they would like to participate in the study were given a consent form after receiving accurate and appropriate information concerning the research project and participation in the research project. Participants were notified that participation in the research project was voluntary and that no negative consequences would be suffered as a result of refusing to participate in the research project. Participants were also notified that they could withdraw from the study at any time they wished to. To ensure that the ethical principle of privacy was adhered to, the researcher conducted interviews on an individual basis with the door closed in a room allocated to the research at the NGO. Privacy was also ensured by giving each participant a unique pseudonym instead of using their names. There was a potential foreseeable minimum risk of harm, which was minor and may have arisen when caregivers discussed the challenges they experienced when caring for children with disabilities at NGOs. It was planned for participants who became distressed and required further psychological intervention to be referred to a clinic closest to them by the authors. Caregivers were assured that they did not have to be anxious regarding their participation in the study. They were assured that anonymity would be guaranteed and that their participation or nonparticipation in the study would not affect their employment. Audio tapes were identified by using codes and not names of participants. All transcripts and voice recordings were kept in a locked cabinet in the main author’s office. Only the main author has access to the transcripts and voice recordings. All computer files containing records are password protected. The University of South Africa’s Policy on Research Ethics stipulates that data be retained for a minimum period of five years. Data will therefore be retained for five years. Thereafter documents will be shredded, and voice recordings will be deleted.

## Findings

Demographic information about the caregivers is firstly presented in Table 1, which is followed by a discussion of the challenges experienced by caregivers who care for children with disabilities at NGOs.

**TABLE 1:** Demographic information of caregivers.

Pseudonym	Gender	Age (years)	Role	Employment period	Level of education
Amy	Female	48	Caregiver	1 year	Grade 11
Bridget	Female	51	Caregiver	7 year	Grade 12
Charmian	Female	37	Caregiver	4 year	Grade 11
Dora	Female	40	Caregiver	6 year	Grade 11
Emma	Female	40	Caregiver	3 year	Grade 11
Farah	Female	48	Caregiver	3 year	Grade 11
Gemma	Female	37	Caregiver	11 year	Grade 9
Hanna	Female	47	Caregiver	3 year	Grade 8
Isabel	Female	52	Caregiver	2 year	Grade 10
Jane	Female	37	Caregiver	8 months	Grade 11

## Themes

The authors generated six themes that represented the challenges and experiences of caregivers of children with disabilities at NGOs, which were: (1) initial impressions, (2) rendering care, (3) stress, (4) lack of outside support, (5) coping and (6) poor community recognition.

### Initial impressions

Caregivers reported their initial reaction to the caregiving context to be shock, sadness and fear. Caregivers were not prepared for what to expect when they first entered the caregiving context:

‘I came here knowing nothing, but right now I am OK. I want to know everything on how to care for the disabled children … At first I was very stressed and felt very sad for these kids.’ (Emma, female, 40 years)

‘I was so shocked the first time I started working here. I question myself as to what am I doing in this place. As time went on I realised their children are just like any other children. Some can’t talk at all, some can’t talk properly and some only respond with their hands.’ (Amy, female, 48 years)

‘I feel so bad the first time I came to this place. It was very tough for me to see children like this for the first time. I was scared.’ (Bridget, female, 51 years)

### Rendering care

Most caregivers expressed challenges relating to their main role, which was to render care to children with disabilities. These challenges included challenges related to bathing, dressing, feeding, positioning and stimulation of their care recipients:

‘Aye … medication. The medication confuses me, but I want to try my best. I don’t do it and I want to do it, but it is very difficult.’ (Bridget, female, 51)

‘There are children here who are wearing diapers. I always tell him that if he doesn’t tell me he wants to go to the bathroom, that I will beat him.’ (Bridget, female, 51 years)

‘Because these children are different. Sometimes we don’t know how to treat them, we don’t know how to care for them and we don’t know how to help them.’ (Charmian, female, 37 years)

### Stress

Caregivers experienced stress, as they were not equipped with the relevant skills and knowledge to render care to their care recipients:

‘I sometimes stress a lot. These disabled children makes me very stressed. Some of these children can beat you. When I’m stressed here my head pains.’ (Isabel, female, 52)

‘I just experienced some parents are not satisfied. They like bad mouthing us caregivers. It’s the experience I have. I sometimes feel stressed and tired, but that stress I didn’t take at home.’ (Farah, female, 48 years)

‘It is the parents that [cause] me stress. They often tell me I am lying about their child’s condition and that their child can do more than I say they can. They often make me cry. It is so painful when the parent say that.’ (Hanna, female, 47 years)

### Lack of outside support

Caregivers needed external support from governmental services and professional services to aid their experience and debrief, which was currently not provided to them. Caregivers narrated that they received support from other caregivers at the NGO and NGO managers; however, they did not get any support from outside the NGO, which they reported to need:

‘No, I don’t get the support I need. For now, they don’t send me when I need support. I don’t know, maybe next time when I need help … yes. If I have challenges, it is the managers who support me.’ (Farah, female, 48 years)

‘No support. We need someone who can counsel us. Sometimes we get a lot of stress.’ (Hanna, female, 47 years)

### Coping

Caregivers experienced difficulty coping with all of the challenges they experienced. Some caregivers made use of maladaptive coping strategies to the extent of taking pills and consuming alcohol in order to be able to sleep and cope with their stressors:

‘I would go and sit in the toilet and drink warm water. When I go home, I would drink alcohol.’ (Bridget, female, 51)

‘I don’t cope; I just buy pills, Disprin, and drink it. I get a lot of headaches. Lots of headaches.’ (Hanna, female, 47 years)

### Poor community recognition

Caregivers did not believe the communities in which they worked recognised the valuable work they were doing at the NGOs. One caregiver stated that people in the community had stigma towards children who were disabled. Caregivers also reported that people in the community did not know and did not understand what they did at the NGO. Community members also did not understand why caregivers preferred to care for children who were disabled:

‘No, they didn’t know what we do here. Some of them ask me what kind of a crèche is it where you work, and I tell them. Others talk about the money and say I do this job only for the money. They not interested in the job I do, they only interested in how much money I earn.’ (Farah, female, 48)

## Discussion

Any person providing acts of nurturing or attending to someone who is in need of such services can be referred to as a caregiver. The act of caregiving echoes the uniqueness of the caregiving role that entails providing emotional support, support with health and medical care, support with basic activities that need to be performed on a daily basis and referrals to relevant medical team members when the need arises (Schulz & Edin 2016). Caregivers could be formal or informal. What distinguishes the two categories of caregivers are the skills and knowledge of the caregiver and remuneration for caregiving services they receive. Formal caregivers are considered to be equipped with the required skills and knowledge to render care to care recipients (Musich et al. 2018). This study identified challenges experienced by caregivers at NGOs caring for children with disabilities.

A majority of caregivers in the study reported their essential caregiving role to be that of providing basic care to children with disabilities. Basic caregiver roles revealed in the study concurs with Schulz and Edin (2016), who regarded the caregiving role as being diverse. Caregivers take on miscellaneous tasks, which makes their role encompass numerous activities. Similar to findings from the current study, Coetzee (2016) mentions caregiving tasks to include bathing, dressing, feeding, diaper changing, medication management and stimulation of care recipients. Providing care to children with severe disabilities is a complex task because of the nature of disabilities these children present with. Most caregivers in the study mentioned numerous challenges related to their specific caregiving role, which could be a result of inadequate training they receive. Bosch (2015) found that most caregivers in rural areas start rendering care to care recipients without having undergone any training. A study by Burgdorf et al. (2019) found that 93% of caregivers providing care to care recipients have never received training to carry out their caregiving role. Elkins and Rustin (2019) conducted a study on caregiver training needs at two different caregiver conferences and found that most caregivers required more training on health issues and use of resources. Training in areas such as diaper changing, potty training, bathing and transferring was also expressed as a need.

Furthermore, the study by Elkins and Rustin (2019) revealed that caregivers also needed training on the conditions that care recipients present with and how to interact with care recipients. Caregivers also expressed the need for training on how to improve physical interactions with their care recipients and how to exercise patience, compassion and kindness. Bosch (2015) highlights the importance of providing training to improve the skills and knowledge of caregivers. Caregiver training enhances the quality of life of caregivers and improves the quality of care that care recipients receive. Caregiver training also improves the problem-solving skills of caregivers and decreases the negative effects of caregiving. Because of the lack of resources and availability of rehabilitation specialists, many children with disabilities do not receive the appropriate care they need.

The Department of Social Welfare of the Ministry of Gender, Children and Social Protection and UNICEF Ghana (2020) developed a training manual for children with disabilities that outlines the basic training that caregivers of children with disabilities require. Included in this basic training are child growth and development, types of disabilities, children’s rights, categories of caregivers, basic needs of children, quality of care of children with disabilities and self-care for caregivers of children with disabilities.

From what was revealed in this study, it is evident that the caregiver role is one that carries with it a high level of responsibility. Zarit (2004) argues that those who are in need of caregiving services and do not receive them have lower life expectancies. Numerous authors concur that without the services of caregivers, the burden on the health care system would be higher as more individuals would require hospitalisation or placement in rehabilitation and care facilities (Kutner & Kilbourn 2009; Northouse, Katapodi, Schafenacker & Weiss 2012; Porter, Keefe, Garst, McBride & Baucom 2008).

All caregivers reported that their caregiving role entails providing basic care to children with disabilities at the NGO where they are employed. The basic care caregivers provide to the children include: (1) bathing, (2) feeding, (3) nappy changing, (4) stimulation, (5) potty training and (6) giving medication. Regarding medication, one caregiver expressed her intense fear of giving children their medication; however, she did have a desire to learn more about medication so that giving children their medication could also be added to her caregiving role. Taking children for clinic visits was a role stated by two participants in the study, as parents were unable to take children to the clinic during the clinic operating hours. One caregiver reported performing general caregiving roles for the children as well as basic roles that were usually performed by support staff and cleaners. These general roles included cooking for the children and cleaning of the NGO premises. Performing general and specific roles made it very difficult to manage her time. Caregivers caring for children with intellectual disabilities reported an additional role, which was the role of teaching children basic concepts. Caregivers were responsible for the emotional and physical support for those individuals who were unable to take care of themselves because of physical, emotional and cognitive impairments (Geiger 2012). Schulz and Edin (2016) report that the caregiver roles vary and change over time based on the changing needs of the care recipients.

A frequently performed role of caregivers is that of managing a care recipient’s medication. Often, caregivers do not possess the skills and knowledge of medication management prepared to manage intricate medication schedules of those they render care to (Look & Stone 2018). More than half of the caregivers in the study have the role of medication management. Look and Stone (2018) contend, ‘Medication management is complex and involves many physical and cognitive activities for caregivers’. Despite the numerous challenges caregivers experienced with medication management, they still did express the desire to learn so that they could optimally fulfil this role, as depicted in the excerpt above.

According to Ogle, Cooke and Brandt (2014), caregivers may also benefit from being involved in programs aimed at educating them about the medication regimes of the children they render care to. Tools such as notes, calendars, reminders and checklists could be used to enhance the effectiveness of caregiver medication management, as these will assist them to keep track of the various medications and the times when medications need to be administered (Ogle et al. 2014).

Children arrive at the NGO early in the morning and leave late in the afternoon. Caregivers therefore have an added responsibility of having to take their care recipients to the local clinic for their medical care. A study conducted in South Africa by Mafune, Lebese and Nemathaga (2017) reports an additional role of the caregiver, where the caregiver has to often take their care recipients to the clinic for their general check-ups and medications. Their study also found that nurses at the clinic were furious with caregivers who were not compliant with the care recipients’ medication regimes. These are in line with the findings of this study.

Caregivers in the current study described their first impressions and initial reactions as shock or feeling intensely overwhelmed, as they were not expecting to see children who were severely disabled. Most caregivers reported never receiving any training before assuming their caregiving roles. Some caregivers reported that they were sent on training only after starting their jobs or received ‘on the spot’ training from other caregivers or given instruction by NGO managers on what to do. Similar to the current study, Mapira, Kelly and Geffen (2019) found that most community workers had to assume duties without having undergone the necessary training. The results of the current study are also consistent with a study by Burgdorf et al. (2019:835), published in the *JAMA Internal Medicine Journal*, which revealed that more than three-quarters of caregivers are actively performing their caregiving roles without having undergone any training. A positive first impression is one of the factors that determine caregiver preparedness (Alvariza, Häger-Tibell & Holmet 2020). Caregiver preparedness is described as the perceived readiness of caregivers to undertake the caregiving role that includes the provision of physical and emotional support to those in need (Schumacher et al. 2008). Ferrell and Mazanec (2009) suggest that there exists a strong relationship between caregivers’ preparedness and caregiver burden.

Caregivers are responsible for creating favourable environments that facilitate the process of assisting these children with disabilities to whom they render care. Caregivers, however, face many challenging conditions, which makes it difficult for them to optimally fulfil their caregiver role. Most caregivers mention numerous challenges related to their specific caregiving role. Caregivers find it difficult to handle some children, especially those who display problematic behaviours. Two caregivers stated that they beat children who do not behave accordingly. Giving children medication is another challenge some caregivers reported experiencing, as the dosage of medications and the times given must be precise. Caregivers render care to children with various disabilities who each have their own specific needs, which makes it difficult for caregivers to know how to help children with these different disabilities. There are varieties of factors that pose challenges to caregivers who care for children with disabilities. Amongst these factors are the physical condition of the caregiver, level of knowledge of the caregiver, nature of the child’s disability, age of the caregiver and financial cost of caregiving (Ndadzungira 2016).

The caregiver role is physically and emotionally demanding for caregivers, which often results in them experiencing high levels of stress. Caregivers take on great responsibilities when they care for children who are disabled. Children with disabilities are not easy to care for, as they require intricate care to perform activities of daily living, even the most basic activities such as bathing and dressing. Caregivers thus experience many stressors but are not able to cope with the negative effects of the caregiving role. Because of the requirements of their work, caregivers who care for children with disabilities experience many stressors resulting in the deterioration of caregivers’ physical and psychological well-being. Findings from the study are consistent with literature that identifies anxiety, depression and insomnia as psychological effects of stress (Cora et al. 2012). Stress could also have physical manifestations such as body aches and pains. Research shows that caregivers who are unable to cope with the stress they experience have a lower life expectancy than caregivers who can manage their stress in a healthy manner (Braun et al. 2007). Programmes and interventions aimed at helping caregivers should focus on teaching caregivers skills to deal with the problems the care recipients present with. Equipping caregivers with skills and knowledge to optimally perform their caregiving role could help in drastically reducing the stress caregivers experience.

Findings of this study revealed that some participants self-medicate using over-the-counter painkillers whilst others drink alcohol to help them cope with the stress of their caregiving job. The findings of this study concur with previous research. A study by Rahmani et al. (2019) found that majority of male caregivers used coping strategies that were problem-focused as opposed to the majority of female caregivers, who made use of maladaptive coping strategies. This finding of the study by Rahmani et al. (2019) is in line with the findings from the current study where all caregivers were female caregivers who made use of maladaptive coping strategies. Caregivers reported maladaptive coping strategies to deal with stress that occurred because of their caregiving role. Prior studies have identified stress as a negative impact of caregiving (Theofilou 2012). There is a crucial need for all stakeholders involved with NGOs for children with disabilities to address the adverse effects caregivers experience.

The study revealed that caregivers are receiving inadequate support from outside. Caregivers narrated that they received support from other caregivers at the NGO and NGO managers; however, they did not get any support from outside the NGO, which they reported needing. Hanna highlighted that the outside support she required was counselling, as her job was stressful. Other caregivers, governmental services and professional services could offer support, as those who come into contact with the caregiver are in a position to provide support. Support caregivers could receive is reliant on individual caregivers’ personal experiences and circumstances. Muller-Kluitsi and Slabberti (2020) support this notion and mention that it is vital to make use of a bottom-up approach when planning on how best to support caregivers, where the challenges and needs of caregivers are understood from the caregiver’s perspective. According to The Alzheimer’s Society of York (2018), caregivers who receive the necessary support have their risks of deteriorating heath or distress reduced:

‘[*R*]egardless of the type of barrier to role recognition, when the caregiver role is not identified, it can be challenging for the caregiver to become informed and empowered to meet his/her own needs or become a partner in the care process.’ (The Alzheimer’s Society of York 2018)

The Alzheimer’s Society of York (2018) further iterates that not recognising the caregivers’ role could set off a negative chain of events that lead to potentially high physical and emotional health risks for the caregiver. The vital role caregivers play is one that should not go unrecognised. Most caregivers in the study feel they do not receive adequate recognition from their communities. Schneider (2020) states caregivers who are not recognised by their community are often demoralised and experience higher levels of stress than caregivers who receive community support and recognition. This could explain the high level of stress and poor coping strategies caregivers in the current study experience. Caregivers are crucial members of the team involved with children with disabilities as they contribute significantly to the South African health care system, particularly the primary level of health care.

## Conclusion

Exploring and describing the challenges of caregivers providing care to children with disabilities at NGOs revealed numerous challenges experienced by caregivers, such as initial impressions, rendering care, stress, lack of outside support, coping and poor community recognition. Caregivers require comprehensive programmes that will empower them to render effective care to children who are disabled. It is of utmost importance that caregivers have knowledge, skills, training opportunities, recognition and support to render optimal care for children with disabilities (The Alzheimer’s Society of York 2018). If caregivers of children with disabilities are empowered to effectively address the challenges they face, the care they provide to their recipients will improve and then children’s physical, emotional, social and functional well-being will be enhanced. Empowering caregivers will be of benefit to children who are disabled as well as the caregivers. The physical and psychological distress caregivers experience as a direct result of their caregiving duties is drastically reduced when caregivers receive adequate support (The Alzheimer’s Society of York 2018).

## Recommendations

Caregivers should be empowered and supported to be confident in their capabilities and believe that their work is positive and beneficial so that they are able to deal with the challenges they face, which will ultimately lead to caregivers providing better care to children with disabilities and caregivers experiencing a better quality of life. Newly appointed caregivers should be provided with training before assuming their caregiving duties. Non-governmental organisations should expose their caregivers to various training programmes on a regular basis. Empowering caregivers will also provide affirmation and validation of the roles caregivers play as well as provide a strong sense of self-efficacy for caregivers, which is paramount to caregivers feeling empowered.
